# How to assess the epidemiology of arrhythmogenic ventricular cardiomyopathy

**DOI:** 10.1002/clc.24203

**Published:** 2024-01-12

**Authors:** Naoya Kataoka, Teruhiko Imamura

**Affiliations:** ^1^ Second Department of Internal Medicine University of Toyama Toyama Japan

To editor:

Liu and collaborators conducted a comprehensive evaluation of the prevailing state of arrhythmogenic ventricular cardiomyopathy (AVC) by scrutinizing a nationwide registry database in China.^1^ Their findings revealed a discernible escalation in the incidence of AVC. It was observed that males afflicted with AVC exhibited a commensurate prevalence of ventricular tachycardia and mortality; however, their prevalence of heart failure was comparatively lower than that observed in females. Several salient concerns have been brought to the fore.

The authors availed themselves of the task force criteria for AVC diagnosis, a framework conceived in the antecedent epoch, antedating the advent of genetic elucidation and cardiac magnetic resonance.^2^ In the current scientific milieu, the extant literature vociferously advocates for molecular genetic testing concomitant with the clinical diagnosis of AVC.^3^ Although the authors ostensibly employed cardiac magnetic resonance imaging and endomyocardial biopsy to ascertain left ventricular involvement, elucidation on the finer nuances of the diagnostic criteria employed would substantiate the veracity of their diagnoses.

The authors probed the prevalence of ventricular tachycardia.^1^ It is pertinent to note that in our antecedent study with an Asian cohort, we posited that patients often manifest ventricular fibrillation, particularly in the nascent phases of AVC.^4^ Do the authors possess arrhythmia data beyond ventricular tachycardia?

In the purview of the authors' investigation, a significant proportion of AVC patients received their initial diagnoses before attaining the age of 20.^1^ In contradistinction, an alternate Asian cohort study posited the zenith of initial AVC diagnoses between the ages of 40 and 50.^4^ How do the authors reconcile this apparent incongruity? It is plausible that familial genetic scrutiny, rather than clinical events such as arrhythmia, served as the impetus for diagnosis in numerous cases within the authors' study.

## Data Availability

Data sharing not applicable to this article as no datasets were generated or analysed during the current study.

## References

[1] Liu ST , Li R , Zheng JP , et al. Epidemiology of arrhythmogenic ventricular cardiomyopathy in China. Clin Cardiol. 2023. 10.1002/clc.24160 PMC10766133 37915277

[2] Marcus FI , McKenna WJ , Sherrill D , et al. Diagnosis of arrhythmogenic right ventricular cardiomyopathy/dysplasia: proposed modification of the task force criteria. Eur Heart J. 2010;31:806‐814. 20172912 10.1093/eurheartj/ehq025 PMC2848326

[3] Corrado D , Perazzolo Marra M , Zorzi A , et al. Diagnosis of arrhythmogenic cardiomyopathy: the Padua criteria. Int J Cardiol. 2020;319:106‐114. 32561223 10.1016/j.ijcard.2020.06.005

[4] Kataoka N , Nagase S , Kamakura T , et al. Clinical differences in Japanese patients between Brugada syndrome and arrhythmogenic right ventricular cardiomyopathy with long‐term follow‐up. Am J Cardiol. 2019;124:715‐722. 31284935 10.1016/j.amjcard.2019.05.067

